# Divergent outcomes of delayed ureteropelvic junction stenosis after renal cyst sclerotherapy: two case reports

**DOI:** 10.3389/fmed.2026.1948851

**Published:** 2026-09-16

**Authors:** Hanchi Yu, Jun Chen, Tianqiang Yu, Hengzi Wang, Zhefeng Xu, Qing Hu, Yue Duan, Jun Fu

**Affiliations:** 1 Department of Urology and Andrology, The Second Affiliated Hospital of Zhejiang Chinese Medical University, Hangzhou, China; 2 Zhejiang Provincial Key Laboratory of Traditional Chinese Medicine, Hangzhou, China; 3 Zhejiang Provincial Key Laboratory of Sexual Function of Integrated Traditional Chinese and Western Medicine, Hangzhou, China; 4 Urology and Andrology Laboratory, The Second Affiliated Hospital of Zhejiang Chinese Medical University, Hangzhou, China; 5 Department of Urology, Zhejiang Rongjun Hospital, Jiaxing, China; 6 Department of Urology, Marine Police Corps Hospital of Chinese People’s Armed Police Force, Jiaxing, China

**Keywords:** case report, complications, hydronephrosis, renal cyst puncture and sclerotherapy, ureteropelvic junction stenosis

## Abstract

Rare long-term complications, such as ureteropelvic junction (UPJ) stenosis, can occur after renal cyst puncture and sclerotherapy and lead to severe hydronephrosis with renal impairment. We illustrate these risks with two examples: in one case, a delayed diagnosis led to severe perirenal fibrosis and renal failure at operation; in the other case, early laparoscopic pyeloplasty preserved renal function. The marked differences illustrate that this complication often has a mysterious onset and long latency, highlighting the importance of early detection and treatment. Clinicians should maintain a high index of suspicion, inform patients of long-term risks before the procedure, and implement long-term follow-up. Regular imaging monitoring can allow early diagnosis and treatment, which reduces the risk of irreversible renal function loss.

## 1 Introduction

Renal cyst puncture sclerotherapy is the accepted minimally invasive treatment that provides reliable symptom relief with a good safety profile (1, 2). However, it is not devoid of risks (1, 3). Although rare, certain late complications can be clinically devastating (3–5). One such complication is ureteropelvic junction stenosis following renal cyst sclerotherapy, which can lead to progressive hydronephrosis and renal failure, ultimately resulting in the loss of kidney function (6, 7). While intraoperative and short-term complications are well documented, the delayed presentation of obstructive uropathy often goes undiagnosed and untreated, as typical symptoms are frequently absent (1, 4).

We present two instructive cases. In one case, a delayed diagnosis accompanied by extensive perirenal fibrosis necessitated nephrectomy. In the other case, prompt laparoscopic pyeloplasty preserved renal function. These results underscore the need for enhanced clinical surveillance for late complications following renal cyst sclerotherapy and the importance of long-term follow-up to prevent such complications (4).

The uniqueness of this case series lies in the starkly contrasting clinical trajectories following the same procedure, directly linked to the timing of diagnosis. We provide a detailed description of the pathophysiological process of delayed UPJ stricture presenting months to years after the use of absolute ethanol—the most common sclerosant currently in use—which has rarely been documented (3, 5). This report adds to the literature by emphasizing that an uneventful early postoperative course does not guarantee freedom from future complications, advocating for a paradigm shift towards mandatory long-term follow-up.

## 2 Case description

### 2.1 Case 1

#### 2.1.1 Patient information

The patient is a 60-year-old male who underwent bilateral renal cyst puncture and sclerotherapy in 2021 at an outside hospital for bilateral renal cysts; whether the symptomatic right-sided cyst was a parapelvic cyst or a cortical cyst could not be definitively determined from the available outside-hospital records. He recovered well, with no immediate or early procedure-related complications. Detailed procedural records from the outside hospital (e.g., total sclerosant volume) were not available; the agent used was absolute ethanol. No relevant family, genetic or psychosocial history was reported.

#### 2.1.2 Clinical findings

Two weeks prior to his admission on 2025.11.03, he developed persistent right-sided flank pain without fever or haematuria. A CT urogram demonstrated significant hydronephrosis with marked dilation of the right renal pelvis and calyces; the right collecting system was not visualised, suggesting possible stenosis at the ureteropelvic junction (Figure 1A).

**Figure 1:**
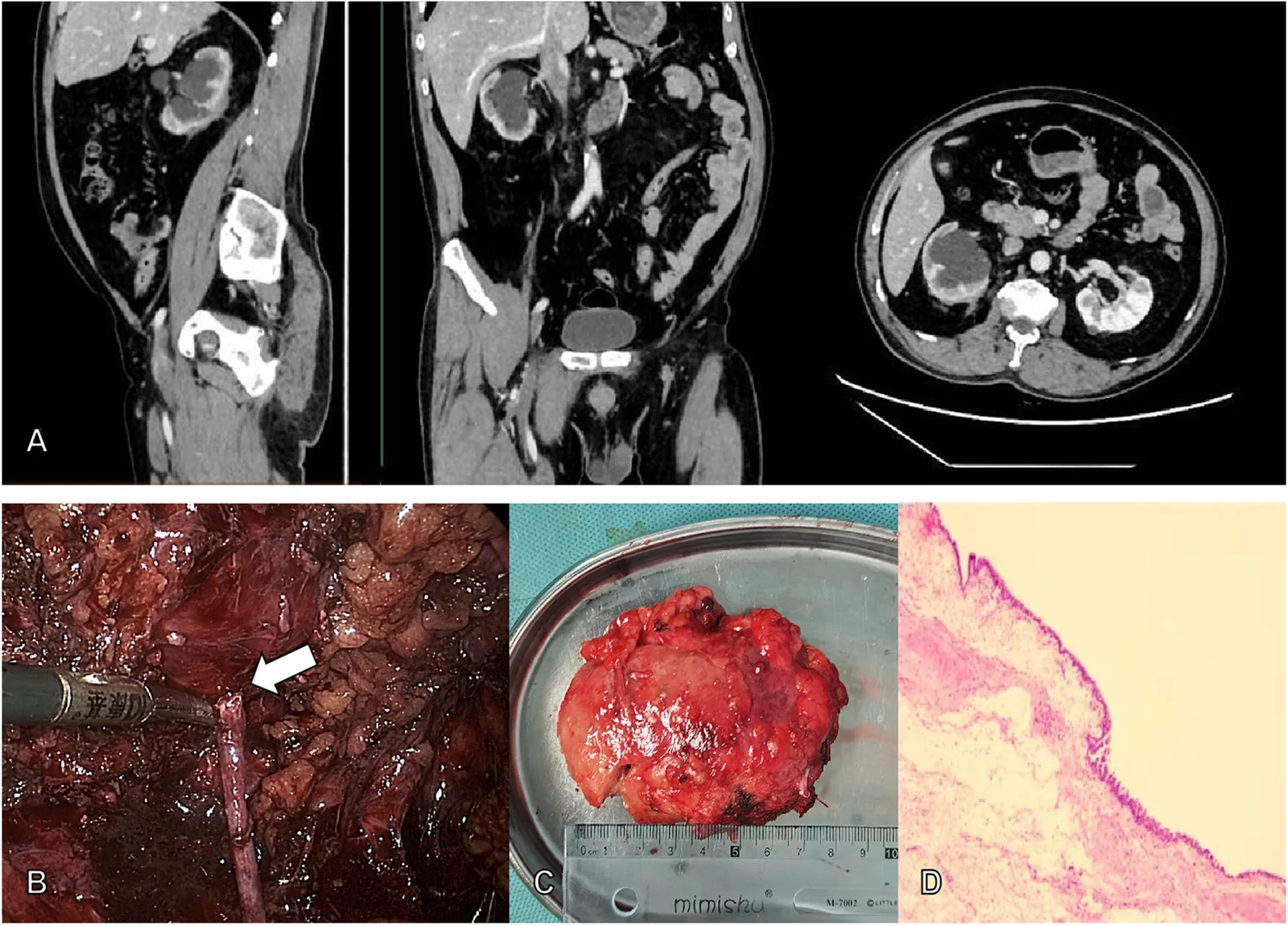
**(A)** CTU image of the stenosis at the ureteropelvic junction following renal cyst puncture and sclerotherapy; **(B)** in case 1, during the intraoperative procedure of planned pyeloureteric reconstruction, it was found that the anastomotic stoma had excessive tension (the arrow in the figure indicates the intended anastomotic site.); **(C)** in case 1, the gross specimen after the operation showed obvious atrophy of the affected kidney; **(D)** the postoperative pathological image of case 1.

#### 2.1.3 Diagnostic assessment

Given the history of right renal cyst puncture and sclerotherapy and the temporal relationship to the procedure, the hydronephrosis was highly suspected to be secondary to UPJ stenosis following sclerotherapy. The type of the treated cyst and the precise puncture trajectory could not be confirmed from the outside-hospital records. After a thorough preoperative evaluation and discussion with the patient, laparoscopic pyeloureterostomy was planned to relieve the obstruction and preserve renal function. Preoperative CT urography showed no contrast excretion from the right kidney (the right collecting system was not visualised) together with marked parenchymal thinning, indicating severely impaired right renal function (serum creatinine 157.3 μmol/L; eGFR 40.8 mL/min/1.73 m^2^). A formal split renal function study was not performed preoperatively, as this examination was not yet available at our hospital at the time. The pre-procedural imaging from the outside hospital could not be retrieved; however, the patient had no symptoms, signs or documented imaging findings of hydronephrosis or UPJ obstruction before sclerotherapy.

#### 2.1.4 Therapeutic intervention

Intraoperative findings were more severe than anticipated (Figure 1B): extreme fibrosis around the renal hilum with hard tissue and loss of normal anatomical structures; the right kidney was markedly atrophied with a thinned parenchyma, consistent with the severely impaired function already suggested by the preoperative CT urography (non-visualisation of the right collecting system). Due to extensive fibrotic adhesions and contractures, attempts to create a tension-free anastomosis between the ureter and the lower renal calyx were unsuccessful, with excessive tension at the anastomotic site predicting a high risk of postoperative restenosis and urine leakage. Because neither pyeloplasty nor ureterocalycostomy was technically feasible, laparoscopic surgery could not achieve the goals of preserving the kidney and ensuring unobstructed drainage, necessitating conversion to open surgery for a right nephrectomy. A formal split renal function study was regrettably not obtained before surgery; in retrospect, it would have allowed the poor functional prognosis to be documented objectively.

#### 2.1.5 Follow-up and outcomes

Pathological examination of the postoperative renal specimen (Figure 1C) confirmed several significant findings in the right kidney (Figure 1D): atrophy of the renal cortex, widespread glomerular atrophy and sclerosis, dilation of the renal tubules, marked dilation of the renal pelvis and calyces with haemorrhage, and interstitial fibrosis with substantial chronic inflammatory cell infiltration – all consistent with hydronephrotic changes due to prolonged obstruction. The perirenal free tissue primarily consisted of extensively hyalinised fibroadipose tissue, with chronic inflammatory cell infiltration, old haemorrhage, and calcification. No foreign-body giant cell reaction or granuloma formation was observed. At the 1-week postoperative follow-up on 2025.11.14, renal function showed no significant decline. The diagnostic and therapeutic timeline for this case is presented in Table 1.

**Table 1:** Timeline of clinical course for case 1.

Date/Period	Event
2021	Underwent bilateral renal cyst puncture and sclerotherapy at an external hospital. Uneventful recovery.
2025.10.20	Developed persistent right flank pain.
2025.11.03 (Day of admission)	CTU revealed significant right hydronephrosis and UPJ stenosis. Admitted for further evaluation. [CREA 157.3 μmol/L, eGFR 40.8 mL/(min·1.73m^2)]
During admission	Laparoscopic pyeloureterostomy was attempted but converted to nephrectomy due to severe perirenal fibrosis and a non-salvageable kidney.
2025.11.14 (Postoperative)	Pathological examination confirmed chronic hydronephrotic changes. Patient recovered and was discharged. [CREA 145.8 μmol/L, eGFR 44.8 mL/(min·1.73m^2)]

### 2.2 Case 2

#### 2.2.1 Patient information

The patient is a 39-year-old man who underwent percutaneous renal cyst puncture and sclerotherapy (Figures 2A,B) at an outside hospital for a “left parapelvic cyst” on 2015.12.17. He recovered well postoperatively with no immediate discomfort. Detailed procedural records from the outside hospital (e.g., total sclerosant volume) were not available; the agent used was absolute ethanol. No relevant family, genetic or psychosocial history was reported.

**Figure 2:**
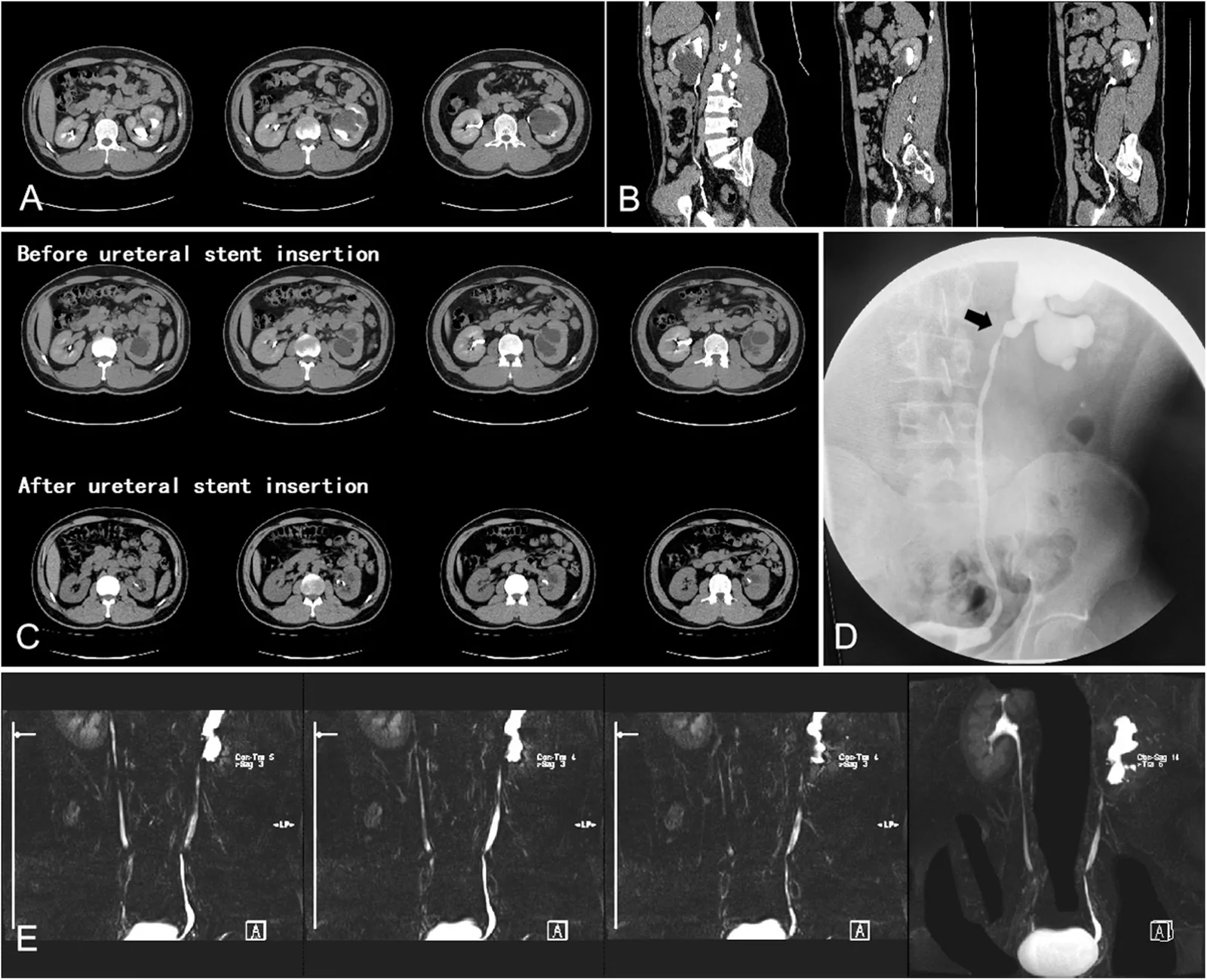
**(A)** Axial CTU images of case 2 before percutaneous renal puncture sclerotherapy; **(B)** sagittal CTU images of case 2 before percutaneous renal puncture sclerotherapy; **(C)** CT images of case 2 before and after ureteral stent placement after percutaneous renal puncture sclerotherapy; **(D)** retrograde pyelography of case 2 after percutaneous renal puncture sclerotherapy, showing the stenosis at the ureteropelvic junction (the black arrow in the figure indicates the stenosis); **(E)** urological MRI images of case 2 during the follow-up after pyeloplasty.

#### 2.2.2 Clinical findings

On 2016.05.03, he developed a distending pain in the left lumbar region. Both CT (Figure 2C) and urogram (Figure 2D) revealed stenosis of the left ureteropelvic junction (UPJ) with secondary moderate hydronephrosis of the left kidney. Preoperative catheterisation improved the hydronephrosis.

#### 2.2.3 Diagnostic assessment

Based on the history of parapelvic cyst sclerotherapy 5 months earlier, the clinical diagnosis was iatrogenic UPJ stenosis. An indwelling ureteral stent provided marked relief of the left hydronephrosis, further supporting the diagnosis. After full discussion with the patient, laparoscopic pyeloplasty was planned.

#### 2.2.4 Therapeutic intervention

Intraoperatively, perihilar fibrosis was limited, the renal pelvis was markedly dilated, and no significant renal parenchymal atrophy was observed. The stenotic segment was excised, and a tension-free pyeloureteral anastomosis was completed.

#### 2.2.5 Follow-up and outcomes

The patient had an uneventful recovery, and his lumbar pain resolved. Follow-up MRI performed on 2016.12.12 showed marked improvement of hydronephrosis (Figure 2E), and the left kidney was successfully preserved. The diagnostic and therapeutic timeline for this case is presented in Table 2.

**Table 2:** Timeline of clinical course for case 2.

Date/Period	Event
2015.12.17	Underwent percutaneous renal cyst puncture and sclerotherapy for left parapelvic cyst. Uneventful recovery.
2016.05.03 (1 week prior)	Developed distending left lumbar pain.
2016.05.10 (Day of admission)	CTU revealed left UPJ stenosis with moderate hydronephrosis.
During admission	Ureteral stent placed temporarily, followed by laparoscopic pyeloplasty.
2016.12.12 (6-month follow-up)	MRI showed marked improvement of hydronephrosis; patient asymptomatic.

## 3 Discussion

Percutaneous aspiration and sclerotherapy (PAS) of renal cysts is a well-established, minimally invasive and effective treatment for symptomatic simple renal cysts (1, 8). Common complications are usually mild and self-limiting, including pain at the puncture site, transient fever and haematuria (9, 10). Severe complications after renal cyst sclerotherapy are reported extremely rarely (1, 11). The existing literature has mainly emphasised PAS's high success rate and low recurrence or a few recognised rare complications, such as malignant transformation of renal cysts after sclerotherapy (4, 10). Reports of UPJ obstruction directly caused by sclerosant extravasation are even rarer, though not unprecedented (12). Earlier studies noted that sclerosant extravasation is a potential risk factor for ureteral stricture (13), and a 1980 report described UPJ obstruction after use of isophendylate as a sclerosant (14). Here, we provide the first detailed description of delayed UPJ stricture presenting months to years after use of the currently most common sclerosant, absolute ethanol, and of the associated pathophysiological process.

In both cases, particularly those involving parapelvic cysts, a minute quantity of absolute ethanol can leak into the adipose tissue surrounding the renal hilum, renal pelvis and upper ureter during insertion or removal of the puncture needle. Ethanol, a potent chemical agent, causes cellular dehydration, protein denaturation, coagulative necrosis and thrombus formation in small vessels (15–18). Direct contact of ethanol with perirenal tissue immediately provokes severe chemical inflammation and tissue necrosis (15, 19). This primary injury then initiates a prolonged, low-grade chronic inflammatory repair process, the pathological hallmark of which is persistent fibroblast proliferation and excessive collagen deposition, i.e., reactive fibrosis (19–21). Progressive accumulation, thickening and contraction of fibrous scar tissue around the renal pelvis forms a rigid “capsule” compressing and wrapping around the UPJ. This leads to luminal narrowing and obstruction of the urinary tract.

In Case 1, we initially attempted laparoscopic repair of the renal pelvis but subsequently proceeded to open surgery. This decision was based on the intraoperative assessment, which accurately reflected the lesion's severity. Two principal factors rendered laparoscopic reconstruction unsuccessful. First, normal anatomical planes were obliterated, and the tissues were leathery and unyielding, preventing safe dissection and mobilisation of the kidney and making a tension-free anastomosis between the lower renal pelvis and the ureter unattainable. Second, the kidney showed marked atrophy and parenchymal thinning, and the right collecting system had not been visualised on preoperative CT urography, indicating severely impaired function; more importantly, the extensive perirenal fibrosis and contracture made a tension-free anastomosis impossible, so that nephrectomy was the only technically feasible option (1, 22). In hindsight, a preoperative split renal function study would have provided objective documentation of the poor functional prognosis. Even if an anastomosis had been attempted, the risk of postoperative stricture and urinary leakage would have been prohibitively high. Nephrectomy was therefore the only rational and patient-centred option. It relieved the flank pain caused by obstruction, removed the source of infection and chronic discomfort, and prevented further complications such as pyonephrosis and stone formation.

These two cases offer important cautions for clinical practice. First, improve preoperative informed consent: although the incidence is extremely low, “delayed UPJ stenosis, progressive hydronephrosis, loss of renal function and even eventual nephrectomy” should be documented explicitly as rare but serious potential risks of PAS, particularly for cysts that are deep or adjacent to the renal pelvis. Second, follow procedural guidelines strictly: use ultrasound or CT for precise localisation to avoid the collecting system and major hilar vessels; obtain cyst fluid after puncture for creatinine analysis to exclude communication with the collecting system; drain the cyst as completely as possible before instilling sclerosing agents; instil a volume less than that aspirated; and deliver the agent slowly and at low pressure to minimise intracystic pressure and leakage (23). Third, long-term follow-up: The principal lesson from these two cases is that an uneventful early postoperative course after PAS does not guarantee freedom from later complications. Because this complication can be delayed and clinically silent, patients may remain asymptomatic until substantial renal impairment has already occurred. Therefore, we strongly recommend implementing a long-term imaging surveillance protocol for all renal cyst sclerotherapy, particularly for parapelvic cysts (22). This approach enables early detection of progressive hydronephrosis during the asymptomatic phase; timely intervention can relieve obstruction, preserve renal function and prevent loss of the kidney.

The present report has several limitations. First, detailed procedural records from the outside hospitals were unavailable, and the pre-sclerotherapy imaging of Case 1 could not be retrieved; consequently, the cyst type (parapelvic or cortical) and the puncture trajectory could not be confirmed. Second, because Case 1 underwent no imaging between sclerotherapy (2021) and presentation (2025), the time of onset of UPJ stenosis cannot be established; “delayed” thus denotes late diagnosis rather than a precisely dated onset. Third, a split renal function study was not obtained preoperatively in Case 1, as this examination was not yet available at our hospital; renal scintigraphy is recommended before salvage surgery when long-standing obstruction is suspected. Fourth, the absence of foreign-body giant cells or granulomas did not reliably distinguish sclerosant-induced fibrosis from idiopathic retroperitoneal fibrosis; the diagnosis rested on the overall clinicopathological picture, chiefly the temporal relation to sclerotherapy and the absence of systemic features of the latter.

Delayed UPJ stricture following percutaneous renal cyst sclerotherapy is a rare but serious complication. It can arise from chronic, progressive perirenal fibrosis due to microscopic leakage of the sclerosant. Urologists should be aware of this risk, adhere to strict procedural protocols, and provide patients with preoperative counselling and appropriate long-term imaging follow-up to enable early and timely detection and treatment.

Data availability statementThe original contributions presented in the study are included in the article/Supplementary Material, further inquiries can be directed to the corresponding author.
